# The role of stabilizing and communicating symptoms given overlapping communities in psychopathology networks

**DOI:** 10.1038/s41598-018-24224-2

**Published:** 2018-04-11

**Authors:** Tessa F. Blanken, Marie K. Deserno, Jonas Dalege, Denny Borsboom, Peter Blanken, Gerard A. Kerkhof, Angélique O. J. Cramer

**Affiliations:** 1 Department of Psychology, University of Amsterdam, Nieuwe Achtergracht 129-B, 1018 WS Amsterdam, The Netherlands; 2 Department of Sleep and Cognition, Netherlands Institute for Neuroscience, Meibergdreef 47, 1105 BA Amsterdam, The Netherlands; 3 Dr. Leo Kannerhuis and REACH-AUT, Houtsniplaan 1a, 6865 XZ Doorwerth, The Netherlands; 4 Parnassia Addiction Research Centre (PARC, Brijder Addiction Treatment), Zoutkeetsingel 40, 2512 HN The Hague, The Netherlands; 5 Sleep Disorders Center MCH, Lijnbaan 32, 2512 VA, The Hague, The Netherlands; 6 Department of Methodology and Statistics, Tilburg School of Social and Behavioral Sciences, Tilburg University, Warandelaan 2, 5037 AB Tilburg, The Netherlands

## Abstract

Network theory, as a theoretical and methodological framework, is energizing many research fields, among which clinical psychology and psychiatry. Fundamental to the network theory of psychopathology is the role of specific symptoms and their interactions. Current statistical tools, however, fail to fully capture this constitutional property. We propose community detection tools as a means to evaluate the complex network structure of psychopathology, free from its original boundaries of distinct disorders. Unique to this approach is that symptoms can belong to multiple communities. Using a large community sample and spanning a broad range of symptoms (Symptom Checklist-90-Revised), we identified 18 communities of interconnected symptoms. The differential role of symptoms within and between communities offers a framework to study the clinical concepts of comorbidity, heterogeneity and hallmark symptoms. Symptoms with many and strong connections within a community, defined as stabilizing symptoms, could be thought of as the core of a community, whereas symptoms that belong to multiple communities, defined as communicating symptoms, facilitate the communication between problem areas. We propose that defining symptoms on their stabilizing and/or communicating role within and across communities accelerates our understanding of these clinical phenomena, central to research and treatment of psychopathology.

## Introduction

Ever since the nineteenth century, definitions of mental disorders were formulated and documented in classification schemes to facilitate communication about, treatment of and research on psychopathology (e.g.^1,2^). Although these classification schemes have contributed to the reliability of psychiatric classification for clinical and research purposes, diagnostic inter-rater reliability has been reported to be (very) low for some diagnostic categories (e.g.^3^), suggesting that their fixed phenomenological boundaries might bypass the complex nature of mental disorders. One prominent example of this complexity is the widespread phenomenon of comorbidity – suffering from multiple mental disorders, either within (i.e., concurrent comorbidity) or at different periods in time (i.e., sequential comorbidity)^4^. More precisely, using current classification schemes, almost half of all people diagnosed with one disorder also meet the criteria for one or more additional disorders in their lifetime^5^. While this complexity is often treated as a nuisance, network theory offers unparalleled opportunities to lay bare the workings of complex phenomena (e.g.^6,7^). In the present paper, we use a novel approach – *overlapping community detection* in psychopathological networks in which symptoms of various disorders interact with one another – to represent and potentially explain the complex and interconnected structure of psychopathology.

The high prevalence of comorbidity challenged the validity of the conceptualization of mental disorders as distinct entities^8^ and gave rise to the *network approach* to psychopathology^9^. According to this approach, mental disorders are conceptualized as networks of interrelated symptoms (represented as nodes) that have direct connections to one another (e.g., insomnia influences fatigue: insomnia - > fatigue). As such, symptoms and their connections are constitutive of a mental disorder (e.g., insomnia may cause fatigue and such relations are what constitute the disorder major depression). The network approach thereby concentrates on the evaluation of symptom-to-symptom interactions, both within as well as between disorders, with the network structure of all psychopathological symptoms representing the *landscape of psychopathology*^10^. Within the psychopathology landscape, the concept of comorbidity then follows from symptom-to-symptom interactions that cut across the ‘borders’ of disorders as originally defined in diagnostic classification schemes such as the DSM^11^. The rapidly expanding network approach to psychopathology appears to provide a promising framework to evaluate clinical phenomena, such as hallmark symptoms^12^, heterogeneity^13^, and comorbidity^9^.

Fundamental to the network approach are ‘bridge symptoms’: specific symptoms that connect multiple disorders and thereby form possible origins of comorbidity^9,11,14^. For example, a potential bridge symptom between major depression (MD) and generalized anxiety disorder (GAD) is “sleep problems” (e.g.^9,15^); that is, developing sleep problems may be the bridge that is crossed when someone, who already suffers from MD, develops GAD, or vice versa. Interestingly, the role of bridge symptoms within a psychopathology network corresponds to other well-studied complex observable networks. In social networks, for example, it is often observed that a network element (e.g., a person) belongs to multiple clusters (e.g., different social groups). Such ‘bridge persons’ are a likely route for spreading gossip due to their membership of various clusters^16^. These similar characteristics of psychopathological networks vis-à-vis other complex, observable networks pave the way for using sophisticated statistical techniques developed for these complex networks. One such technique, that will form the heart of the methodology of the present study, is community detection.

Community detection allows for evaluating large networks (e.g., social media networks, psychopathology networks) and identify communities of nodes (e.g., groups of friends, groups of symptoms)^17^. One community detection algorithm that is particularly applicable to psychopathology is the Clique Percolation Method (CPM), as it allows nodes to belong to more than one community (^18^ applied to, for example, the human interactome, e.g.^19^ social media, e.g.^20^ and scientific publications, e.g.^21^). As such, this approach provides a natural representation of the theoretical stance of the network approach on the fuzzy borders between disorders^9^. Within psychopathology networks, such communities may represent specific ‘problem areas’. These problem areas consist of symptoms that are closely connected to each other and tend to co-occur. The identification of these local problem areas provides novel insights into the interdependency of symptoms on the local (community) structure over and above the interdependencies in the global structure of the psychopathology network.

**Figure 1:**
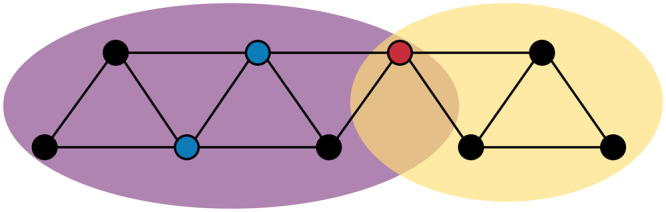
Example of a network with two problem areas: a purple community consisting of six symptoms, and a yellow community consisting of four symptoms. The blue symptoms represent potential stabilizers, while the red symptom represents a bridge symptom that facilitates possible communication between the two problem areas.

For example, Fig. 1 shows two problem areas (purple and yellow) that share a single symptom (red), which corresponds to the notion of *overlapping* communities. Locally, the organization of symptoms within a problem area may yield information on symptoms that *stabilize* the problem area and symptoms that *communicate* with other problem areas^22^. For example, a symptom with many connections to other symptoms in the same problem area is likely to keep, or stabilize, this problem area in a certain state, which can be either healthy or unhealthy (see blue nodes in Fig. 1). Similarly, a symptom with connections across problem areas is likely to *communicate* with other problem areas and might therefore be the origin of their co-occurrence (red node in Fig. 1). Globally, the connections between different problem areas might reveal relevant, novel information on the topology of the overall psychopathology network (e.g., which communities are connected, and how strongly)?

Based on the proposed method, we provide novel formal definitions of two core principles of the network approach to psychopathology. First, *core symptoms* of a problem area are symptoms that have many connections *within* their community and will be coined *stabilizing symptoms*. Second, *bridge symptoms* are symptoms that have many and/or strong connections to other communities, and in some cases even belong to two or more communities, and will be referred to as *communicating symptoms*. In this paper, we show how community detection by means of the CPM method can identify different problem areas of connected symptoms and the structural organization of these problem areas.

## Results

### Global Community Structure

**Figure 2:**
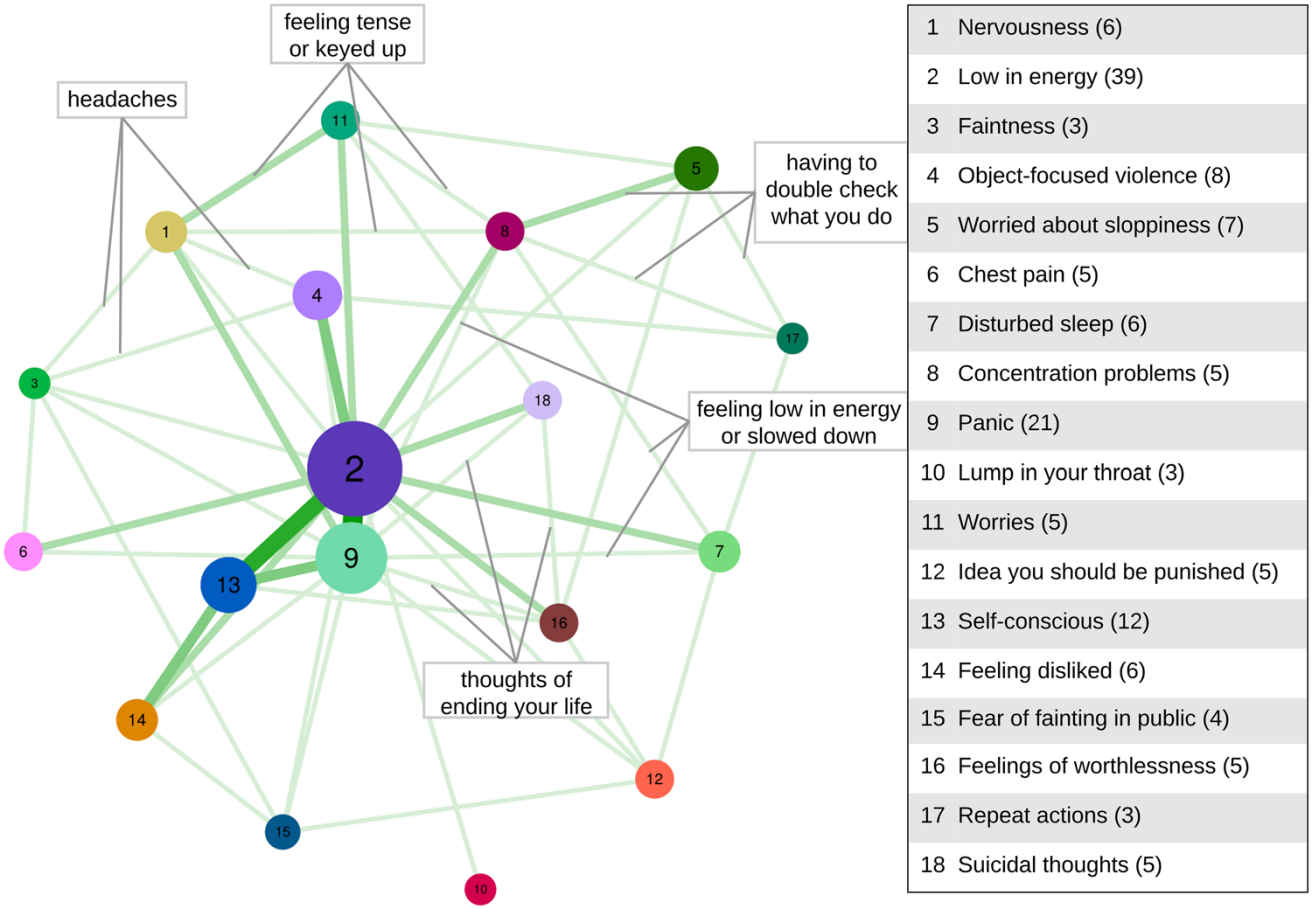
Nodes represent communities and edges correspond to number of symptoms shared, with thicker edges corresponding to more bridge symptoms. Each community is labelled according to its most central (i.e., stabilizing) symptom and its size (i.e., number of symptoms) is depicted between brackets (see legend). The five symptoms depicted in grey boxes are examples of how communities are connected through bridge symptoms.

Based on the *clique-percolation* method, 18 communities were identified in the psychopathology network, with the size of the communities ranging from three to 39 nodes. Appendix A contains a tabulated representation of all communities and their symptoms, ordered by the original SCL-90-R dimensions. Note that we use the SCL-90-R dimension labels whenever we refer to the originally proposed dimensions. Figure 2 shows how strongly these communities are connected via shared symptoms. We labelled the communities (or: problem areas) according to a summary of the most central symptom, i.e., the symptom with the strongest connections to other symptoms within the respective community (e.g., the first community was labelled *Nervousness* according to its most central symptom *nervousness or shakiness inside*). Communities are further denoted by their number (#community number) and symptoms are referred to by the item order in the SCL-90-R (item number). Every community overlapped with at least one other community. The CPM method identified strongest overlap between the following communities: *Low in Energy* (community #2) and *Panic* (#9) share six symptoms, *Low in Energy* (#2) and *Self-conscious* (#13) share five symptoms and *Self-conscious* (#13) shares three symptoms with both *Panic* (#9) and *Feeling disliked* (#14).

Generally, when comparing the resulting community structure (as depicted in Fig. 2) to the SCL-90-R dimensions, a few distinctive global features deserve mention. First of all, while two communities (#3 and #14) consist of symptoms from only a single SCL-90-R dimension, none of them covers all symptoms of a specific dimension. For example, the third community (*Faintness*) consists of merely three symptoms from the Somatization dimension (headaches, faintness or dizziness, and nausea or upset stomach). Second, most communities consist of symptoms from two or more different SCL-90-R dimensions. Although these communities comprise symptoms from originally different dimensions, there seems to be a shared common theme in each community. For example, although the first community (*Nervousness*) covers symptoms from three different SCL-90-R dimensions (Depression, Anxiety, Somatization), all symptoms are related to feeling tense or nervous.

### Local Community Structure

The legend of Fig. 2 lists the 18 communities, labelled according to the symptom that ranked highest on the stabilizing index. These symptoms can thus be expected to have a high impact on nodes within their community or that are most influenced by their community members. For example, item 2 *nervousness or shakiness inside* has the highest stabilizing index in community #1, which consists of symptoms related to feeling tense or nervous. This indicates that feeling nervous or shaky might determine to a large extent whether or not a person also shows several other related symptoms within this community.

In Table 1 we depict the symptoms ranking highest on the communicating index. These symptoms can be expected to play an important role in connecting different problem areas and are thus indicative of bridge symptoms. For example, the symptom *headaches* seems to play an important role in connecting symptoms between the *Nervousness* (#1), *Low in Energy* (#2), *Faintness* (#3), and *Object-focused Violence* (#4) communities. This suggests that symptom activation across these connected problem areas might be predicted by monitoring headaches.

**Table 1:** Strongest communicators that belong to three or more communities.

Symptom (item number)	Communities (# community number)
Headaches (1)	Nervousness (#1), Low in energy (#2), Faintness (#3), Object-focused violence (#4)
Feeling low in energy or slowed down (14)*	Low in energy (#2), Disturbed sleep (#7), Concentration problems (#8), Panic (#9)
Thoughts of ending your life (15)*	Low in energy (#2), Panic (#9), Feelings of worthlessness (#16), Suicidal thoughts (#18)
Hearing voices that other people don’t hear (16)	Low in energy (#2), Object-focused violence (#4), Panic (#9)
Having to double-check what you do (45)	Worried about sloppiness (#5), Concentration problems (#8), Repeat actions (#17)
Feeling hopeless about the future (54)	Low in energy (#2), Worries (#11), Suicidal thoughts (#18)
Feeling tensed or keyed up (57)	Nervousness (#1), Concentration problems (#8), Worries (#11)
Feeling uneasy when people are watching or talking about you (61)	Panic (#9), Self-conscious (#13), Feeling disliked (#14)
Feeling afraid you will faint in public (82)*	Low in energy (#2), Panic (#9), Fear of fainting in public (#15)

^*^These symptoms are also stabilizers.

Note that there are three symptoms that rank high both as communicators and stabilizers (see Table 1). For example, the symptom *feeling low in energy or slowed down* ranked among the highest communicators and stabilizes one of its communities. These symptoms might flag the most decisive symptoms, since they stabilize their community and at the same time communicate with symptoms of other communities. Some overlap between stabilizing and communicating symptoms is hardly surprising, as both these measures depend on the strength and number of connections a symptom has. Yet, as can be seen in Table 1 this overlap is sufficiently low to treat stabilizing and communicating symptoms as different constructs.

### Illustration

Inspecting the local structure offers additional information both on the relations within a community as well as on the communication between communities via so-called *communicating nodes* (i.e., symptoms that have many and/or strong connections to other problem areas). To illustrate this, we focused on the structure of the 5-symptom *Feelings of Worthlessness* community (#16) with mainly symptoms from the depression dimension. This detailed focus has implications for the concept of hallmark symptoms and the study of heterogeneity within this problem area, and also for its comorbidity with other problem areas.

**Figure 3:**
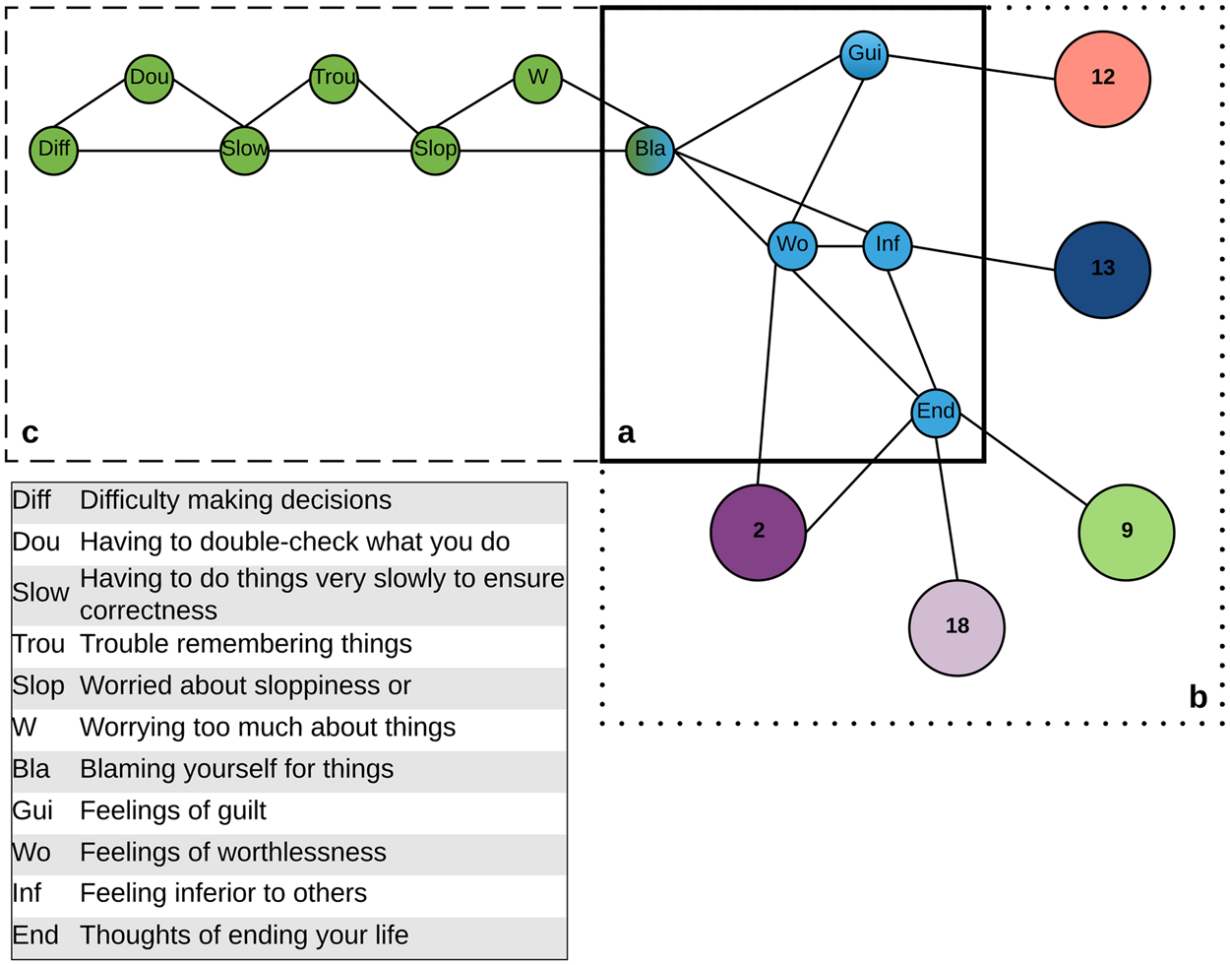
Illustration of (**a**) the local structure of *Feelings of Worthlessness* community (#16), (**b**) its connection to other communities; and (**c**) a symptom-level example of its connection to the community *Worried about Sloppiness* (#5).

First, the stabilizing index of the symptoms in this community reveals what might be its core symptom: *feelings of worthlessness* (item 79) is connected to all other symptoms, i.e., it has a direct connection to *blaming yourself for things, feelings of guilt, feeling inferior to others* and *thoughts of ending your life* (see Fig. 3a). Second, the communicating symptoms of this problem area and their connections to adjacent problem areas, also consisting of symptoms from the depression dimension, reveal possible pathways for the clinical heterogeneity of depression. Figure 3b shows that five additional communities are connected to the *Feelings of Worthlessness* community. For example, the bridge depression symptom *thoughts of ending your life* (item 15) funnels a direct connection to a 5-symptom problem area related to hopelessness (*Suicidal thoughts* #18), such as *thoughts of death and dying* and *feeling hopeless about the future*. Similarly, *feeling inferior to others* (item 41) connects the problem area to another problem area of symptoms on interpersonal sensitivity (*Self-conscious* #13), e.g., *feeling very self-conscious with others* (item 69). Third, the local structure shown in Fig. 3c suggests that the shared symptom of *blaming yourself for things* (item 26) connects depression symptoms (*Feelings of Worthlessness*, #16) to cognitive performance deficits (*Worried about Sloppiness*, #5).

The above presented inspection of the local structure indicates that similar symptoms indeed tend to cluster together and simultaneously points us towards the specific symptoms that funnel the interdependency of problem areas.

## Discussion

In current classification schemes, psychological disorders are defined as distinct entities characterized by hallmark symptoms. In this paper, we evaluated the structure of psychopathology without the theoretical boundaries of these classification schemes, as such allowing for the complex and multi-faceted structure of psychopathology. Specifically, we took a data-driven approach using community detection techniques to study the co-occurrence of symptoms and detect possible problem areas, i.e., communities of closely related symptoms. As such, these problem areas are empirically derived and not theoretically defined.

We applied community detection to a large psychopathological symptom network and identified 18 problem areas. Using these problem areas, we evaluated three complex clinical phenomena that are crucial to our understanding of the development, prevention and treatment of mental disorders: hallmark symptoms, heterogeneity and comorbidity. The results of the current study illustrate how the identified problem areas capture important information on all three phenomena by focusing on the differential role of symptoms within a problem area: *stabilizing* symptoms and *communicating* symptoms. For example, symptoms of the depression dimension decompose into different problem areas. While it is not our objective to rephrase the nosological definition of depression, the problem areas do inform us on what depression symptoms are likely to stabilise smaller problem areas (core symptoms), are more likely to co-occur than others (heterogeneity), or are likely to co-occur with symptoms from other disorders (comorbidity). There are a few promising avenues following from this focus.

First, stabilizing symptoms show many and/or strong connections to other symptoms in the same problem area and could be thought of as the core symptoms of one such problem area. An important next step would be to evaluate whether these symptoms are indeed more prevalent in the designated problem areas. For example, in a problem area consisting predominantly of depression symptoms, *feelings of worthlessness* was a stabilizing symptom. This suggests that this particular symptom is at the core in at least some form of “depression”, implying that for some individuals this symptom may play an important role in triggering and/or maintaining their depression. The importance of this particular symptom is theoretically substantiated in the revised learned helplessness model of depression^23^. While some research based on this model concludes that feelings of worthlessness define one’s vulnerability to develop depression (e.g.^24^), others show that depression samples vary in the reported frequency of this symptom suggesting that it might not be a hallmark symptom^25^. Interestingly, such apparent inconsistencies align with our results: feelings of worthlessness might play a stabilizing role in one, but not all, problem areas that consist of depression symptoms in the reported psychopathology network. It should be investigated whether this symptom indeed has a perpetuating function within the individual network *over time* in people suffering from this constellation of depression symptoms.

Second, communicating symptoms can potentially generate a better understanding of both heterogeneity and comorbidity. Communicating symptoms have many and/or strong connections to other problem areas and, as such, facilitate the communication between problem areas. In the case where two problem areas consist (predominantly) of symptoms that belong to a single disorder as defined in current nosologies, these communicating symptoms relate to the phenomenon of heterogeneity. For example, the peripheral symptom *thoughts of ending your life* bridged two depression-like communities: one characterized by worthlessness and the other characterized by hopelessness. The finding that these symptoms cluster in different depression-like communities points us towards possible patterns of heterogeneity within depression. Depression, as defined by the DSM-V, has shown to be a highly heterogeneous disorder with many different symptom patterns^26^. It would be valuable to assess whether the different depression-like communities correspond to different, clinically observed, symptom patterns. Although the communities identified at a group level cannot be readily extrapolated to the individual level, these symptom patterns, after cross-validation, might offer new leads on what specific symptoms can be identified as a risk factor for infecting a (neighbouring) set of comorbid problems. For example, if an individual suffers mainly from depression symptoms within the Worries (#11) problem area, this individual might be more likely to develop symptoms of the directly related Nervousness problem area (#1; consisting predominantly of symptoms from the anxiety dimension). Conversely, an individual who suffers from depression symptoms in the Feelings of Worthlessness (#16) problem area might have a higher risk to develop or suffer from symptoms of the problem area Idea You Should Be Punished (#12; mostly symptoms on intrusive thoughts)

Alternatively, in the case when the two problem areas consist of symptoms that belong to multiple, supposedly distinct disorders, the communicating symptoms relate to comorbidity. For example, the symptom *blaming yourself for things* belonged to a depression-like problem area characterized by worthlessness and a problem area characterized by cognitive performance deficits. In this particular example, we would hypothesize that *blaming yourself for things* plays a crucial role in patients suffering from not only depression symptoms but also cognitive performance deficit symptoms. It would be important to evaluate whether the identified bridge symptoms indeed play an important role in the co-occurrence of problem areas or disorders in clinical populations. Thus, when adhering to current classification schemes heterogeneity and comorbidity are distinct concepts, while the current framework suggests that they result from the same core principle of communicating symptoms.

Third, we should not limit the investigation of these structures to cross-sectional symptom networks. The study of intra-individual changes in communities of symptoms can point us towards *growth* (i.e., symptoms joining the problem area) or *contraction* (i.e., symptoms leaving the problem area) patterns of specific problem areas that might offer novel starting points for intervention and treatment. Relatedly, these developmental patterns of problem areas could be key to understanding intra-individual characteristics in psychiatry, such as vulnerability and resilience^11,27^. Here, the role of bridge symptoms is crucial: the number and strength of connections of a symptom in one problem area to another problem area increases the probability of this symptom joining the clinical profile of an individual.

### Limitations

A few limitations deserve mention. First, in the current report we studied potential pathways in the landscape of psychopathology in a community sample as we did not aim to draw conclusions about DSM-5 diagnoses. We argue, however, that the results of the current study provide first insights into the structure of the landscape of psychopathology and its constructs in a non-clinical sample. Future investigations should apply this approach to a clinical sample to validate these pathways for clinical populations. Second, while we mostly treated stabilizing and communicating aspects of symptoms as two distinct roles, our results indicated that there is some overlap between stabilizing and communicating symptoms. Future research should focus on further disentangling stabilizing and communicating symptoms and whether overlap between these measures is a relevant finding of its own (e.g., that symptoms ranking high on both measures might be the most decisive symptoms to suffer from). Finally, it should be noted that the identified communities depend on (i) the community detection algorithm, and within the chosen algorithm (CPM), on (ii) the stability of the input network and (iii) the chosen parameters for the clique size k and the intensity threshold I. First, given our goal to further study the complex and sometimes partly overlapping structure of psychopathology, the possibility for a node to belong to multiple communities at once (i.e., overlapping communities), and the absence of strong assumptions about the size and form of local substructures was dominant in selecting an algorithm and resulted in our choice for CPM. Second, to ascertain robustness of the identified communities based on the input network, we assessed the stability of the estimated network. The input network was stable in terms of the strength and number of connections a node has, which is particularly important for the CPM in identifying communities. Third, to select the parameters k and I, we followed the proposed guidelines of Farkas *et al*.^28^ to obtain the richest community structure possible. It is important to note that different communities can be found by changing these parameters. While this can be considered a limitation of the approach, we also believe that this can be a tool to inspect different properties of the psychopathology landscape. For example, by increasing the minimal clique size, one would obtain larger communities and thereby possibly gain more insight into the larger, global structure of psychopathology. Similarly, by increasing the intensity threshold, fewer but more strongly connected communities will be derived. If one aims to inspect the local structure on a more detailed level, lowering the intensity threshold will reveal more, and larger communities. Taking these things into account, we do not propose that problem areas should be interpreted as existing entities. Rather, we argue that applying these techniques can reveal substructures at different levels of symptom networks and allow us to relate them to the role of individual symptoms.

### Concluding Remarks

The identification of stabilizing symptoms comes with great promise, as its state is informative with respect to the state of the other symptoms in its problem area. For example, our results suggest that people with strong feelings of worthlessness are likely to blame themselves for things and experience feelings of guilt (and vice versa). Because of its many strong connections, one is intuitively inclined to interpret the stabilizing symptom ‘feelings of worthlessness’ as a target for intervention programs. The potential success of such an intervention is, however, strongly dependent on the specific nature of the symptom itself (i.e., the symptom itself must be responsive to treatment^29^) and the temporal order of activation in relation to its neighbours (i.e., the symptom must be a cause rather than an effect of its neighbours). Thus, the viability of treating a stabilizing symptom must ultimately be determined longitudinally. Nevertheless, the identification of stabilizing symptoms in cross-sectional data does point us towards potential hubs of the psychopathological system that funnel more symptom activation (either as cause or effect) within a local structure than others.

In sum, the network approach to psychopathology has paved the way for novel techniques to study the interdependency of symptoms as a complex network. To date, however, it was not possible to accommodate the idea of bridge symptoms that connect multiple problem areas. The present study is the first to apply overlapping community detection to psychopathology and conclude that the identification of overlapping communities of symptoms points us towards novel research lines into possible pathways between and within problem areas in psychopathology. In addition, the present study has complemented the network analysis toolbox of clinical psychology and psychiatry by providing novel definitions of communicating and stabilizing roles of symptoms in the psychological landscape.

## Method

### Participants

For the current study, we used data of a study conducted by G. A. Kerkhof and previously reported on in Kerkhof^30^. As explained in Kerkhof (2017), 2089 participants were sampled from an ISO 26362-certified online research panel of Motivaction (https://www.motivaction.nl/en/research-method/online-market-research/online-market-research). When participants enter this panel, they sign an active consent informing them (amongst other things) that their anonymized data can be used for scientific purposes. Participants were recruited from different areas in The Netherlands and the community sample was stratified by age (ranging from 18 to 70, *M* = 48.5, *SD* = 14.0) and gender (49% men and 51% women). The data was collected in accordance with relevant guidelines and regulations and ethical approval was obtained by the Faculty Ethics Review Board (FMG) of the University of Amsterdam.

### Symptom Screening

The Symptom Checklist 90 (SCL-90-R) is a 90-item self-report symptom inventory designed to screen for a broad range of psychological problems. Participants were asked to rate all of the 90 items (see Appendix A) for the last week, including today, on a five-point Likert scale of distress, ranging from *not at all*^1^ to *extremely*^5^. The items can be combined into eight symptom scales: Depression, Anxiety, Agoraphobia, Sleep difficulty, Somatization, Interpersonal sensitivity, Acting-out hostility, and Cognitive-performance deficits. These scales, plus nine unscaled items, can be combined into a total score ranging from 90 to 450, with higher scores indicating more severe symptom levels. The SCL-90-R scores in the current study sample ranged from 90 to 384 (*M* = 124.8, *SD* = 41.8), and 33% of the participants (*N* = 695) scored above the norm score of 123.

### Statistical analyses

#### Network estimation

We estimated a sparse Gaussian network based on the polychoric correlations between the responses to the SCL-90-R items using the *glasso*-method implemented in the R-package *qgraph*^31^. Glasso estimates partial correlations between each pair of variables conditioning on all other variables. As such, we can interpret the network as depicting conditional dependence relations: an edge between two symptoms A and B indicates that these symptoms are conditionally dependent, given all other symptoms in the network. To decrease the number of spurious partial correlations, Glasso uses the regularization technique Least Absolute Shrinking and Selection Operator (LASSO^32^). The LASSO technique utilizes a tuning parameter that controls the sparsity of the estimated network by pushing small edge weights (i.e., polychoric correlations) to zero. The Extended Bayesian Information Criterion (EBIC^33,34^) is then used to select the best fitting regression function. To check the robustness of the estimated network we performed additional network stability checks using the R-package *bootnet*^35^ and appended the results as supplementary material (see Supplementary Methods) of this article.

#### Clique Percolation Method

The CPM algorithm aims to find *percolation clusters* (*communities*) using the notion of *k*-cliques: complete subgraphs of *k* nodes (i.e., a fully connected subgraph of *k* nodes). Two cliques are *adjacent* when they share all but one (*k-*1) nodes. A *k*-*clique percolation cluster* is then defined by “the maximal set of *k*-cliques that can be reached from each other via a set of *k*-clique adjacency connections”^28^. The CPM algorithm has been extended to incorporate edge weights in identifying the communities. This algorithm, called the Clique Percolation Method with weights (CPMw), includes a *k*-clique into a community when its intensity *I* exceeds a fixed threshold^28^. The intensity of a *k*-clique is the geometric mean of the edge weights. Thus, a community according to the CPMw algorithm is the maximal set of *k*-cliques that are *k*-clique adjacent and have intensities above the threshold. Hence, the communities are dependent on the number of nodes *k* in a clique and the intensity threshold *I*.

The CPM algorithm is implemented in the program CFinder^36^. Using the edge list of the Gaussian graphical model estimated on the SCL-90-R data, we determined, for each *k*, the optimal intensity threshold *I* to obtain the richest community structure. Specifically, starting with an intensity threshold equal to the largest edge weight, and then lowering *I* to the point that a giant cluster emerges is called the *percolation transition*. The richest community structure is found at values of *I* just above this transition. In this way, the threshold is high enough to prevent a giant cluster that would obscure the details of smaller communities, and low enough to prevent a large number of separate *k*-cliques^28^.

For each fixed *k* (i.e., *k* = 3, 4, 5, 6), we determined the optimal threshold according to these principles^28^ by (i) lowering the intensity threshold, first set equal to the largest edge weight, by steps of 0.1 until cliques of size *k* emerged; (ii) then we further lowered *I* by smaller steps of 0.01 until a giant cluster emerged, i.e., the percolation transition. Subsequently, (iii) we increased *I* by steps of 0.001 to find the values for *I* just above the percolation transition. After the identification of the optimal *I* value for each *k* separately, we compared the *k* parameters in terms of the broadness of their community size distribution at their optimal *I*.

The initial intensity threshold was set equal to the largest edge weight of 0.48. The optimal threshold for *k* = 3, 4, 5, 6 was equal to 0.099, 0.0695, 0.044, and 0.01, respectively. We then chose *k* = 3 as it had the broadest community structure, i.e., communities of different sizes.

### Local Structure Analysis

To investigate the local structure of the communities, we analyzed the edge weights of connections nodes have *within* and *between* their communities. For the detection of *stabilizing* nodes, we summed the absolute edge weight values a given node has *within* its community (stabilizing index). For the detection of *communicating* nodes, we summed the strength of connections a given node has *between* its communities (communicating index). Notably, these indices are based on commonly used centrality measures in the network literature^37^ usually applied to evaluate the role of symptoms on the global level (i.e., the network as a whole). However, when one assumes that there are meaningful sub-structures within a network on a local level, using centrality measures at the global level does not suffice to identify the symptoms that play an important role at these local levels. Thus, while these metrics are essentially the same, they are applied at different levels. Note that this could imply that symptoms ranking highest on global centrality (taking into account all symptoms at once) might not be the same as the symptoms that rank highest on local centrality (taking only symptoms in the local sub-structure into account).

## Electronic supplementary material

Supplementary Materials
