# Supplementary material for: The role of stabilizing and communicating symptoms given overlapping communities in psychopathology networks
Source: Sci Rep. 2018 Apr 11;8:5854. doi: 10.1038/s41598-018-24224-2 (PMC5895626; doi:10.1038/s41598-018-24224-2)
Supplement: Supplementary file 1 — Supplementary Materials [file 41598_2018_24224_MOESM1_ESM.pdf]

The role of stabilizing and communicating symptoms given overlapping communities in psychopathology networks

Tessa F. Blanken[a,b,1,*]

Marie K. Deserno[a,c,1]

Jonas Dalege[a]

Denny Borsboom[a]

Peter Blanken[d]

Gerard A. Kerkhof[a,e]

Angélique O. J. Cramer[f]

[a] Department of Psychology, University of Amsterdam, Nieuwe Achtergracht 129-B, 1018 WS Amsterdam, The Netherlands
[b] Department of Sleep and Cognition, Netherlands Institute for Neuroscience, Meibergdreef 47, 1105 BA Amsterdam, The Netherlands
[c] Dr. Leo Kannerhuis and REACH-AUT, Houtsniplaan 1a, 6865 XZ Doorwerth, The Netherlands
[d] Parnassia Addiction Research Centre (PARC, Brijder Addiction Treatment), Zoutkeetsingel 40, 2512 HN The Hague, The Netherlands
[e] Sleep Disorders Center MCH, Lijnbaan 32, 2512 VA The Hague, The Netherlands
[f] Department of Methodology and Statistics, Tilburg School of Social and Behavioral Sciences, Tilburg University, Warandelaan 2, 5037 AB Tilburg, The Netherlands
[1] These authors contributed equally to this work.

Supplementary Material

Appendix A. Overview of the SCL-90 items and their associated communities and dimensions.

Supplementary Methods. Network Stability

Appendix A. Overview of the SCL-90 items and their associated communities and dimensions

SCL-90 items and dimensions			communities																	
			#1	#2	#3	#4	#5	#6	#7	#8	#9	#10	#11	#12	#13	#14	#15	#16	#17	#18
Depression	3	Repeated unpleasant thoughts that won't leave your mind	♦										♦							
	5	Loss of sexual interest or pleasure *																		
	14	↔ Feeling low in energy or slowed down		(♦)					♦	♦	♦									
	15	↔ Thoughts of ending your life		♦							♦							♦		(♦)
	19	Poor appetite									♦									
	20	Crying easily		♦								♦								
	22	Feeling of being trapped or caught		♦																
	26	Blaming yourself for things					♦											♦		
	29	Feeling lonely		♦																
	30	Feeling blue		♦									♦							
	31	Worrying too much about things					♦						(♦)							
	32	Feeling no interest in things		♦																
	51	Your mind going blank		♦																
	54	↔ Feeling hopeless about the future		♦									♦							♦
	59	Thoughts of death or dying																		♦
	79	Feelings of worthlessness		♦														(♦)		
Anxiety	2	Nervousness or shakiness inside	(♦)																	
	17	Trembling	♦								♦									
	23	Suddenly scared for no reason									♦									
	33	Feeling fearful									♦									
	39	Heart pounding						♦												
	57	↔ Feeling tensed or keyed up	♦							♦			♦							
	72	Spells of terror or panic	♦								(♦)									
	78	Feeling so restless you couldn't sit still *																		
	80	The feeling that something bad is going to happen to you																		♦
	86	Thoughts and images of a frightening nature									♦			♦						
Agoraphobia	13	Feeling afraid in open spaces or streets									♦									
	25	Feeling afraid to get out of your house alone									♦									
	47	Feeling afraid to travel on buses, subways or trains									♦									
	50	Having to avoid certain things, places, or activities because they frighten you									♦									
	70	Feeling uneasy in crowds such as shopping or at movies									♦				♦					
	75	Feeling nervous when you are left alone										♦								
	82	Feeling afraid you will faint in public		♦							♦						(♦)			

SCL-90 items and dimensions			communities																	
			#1	#2	#3	#4	#5	#6	#7	#8	#9	#10	#11	#12	#13	#14	#15	#16	#17	#18
Sleep difficulty	44	Trouble falling asleep							♦										♦	
	64	Awakening early in the morning							♦											
	66	Sleep that is restless or disturbed		♦					(♦)											
Somatization	1	↔ Headaches	♦	♦	♦	♦														
	4	Faintness or dizziness			(♦)												♦			
	12	Pain in heart or chest						(♦)												
	27	Pains in lower back		♦																
	40	Nausea or upset stomach			♦			♦			♦									
	42	Soreness of muscles		♦																
	48	Trouble getting your breath		♦				♦												
	49	Hot or cold spells							♦											
	52	Numbness or tingling in parts of your body		♦				♦												
	53	A lumb in your throat										(♦)								
	56	Feeling week in parts of your body		♦																
	58	Heavy feelings in your arms or legs		♦																
Interpersonal sensitivity-mistrust	6	Feeling critical of others		♦							♦									
	7	The idea that someone else can control your thoughts		♦																
	8	Feeling others are to blame for most of your troubles		♦											♦					
	18	Feeling that most people cannot be trusted		♦																
	21	Feeling shy and uneasy with the opposite sex													♦					
	34	Your feelings being easily hurt														♦	♦			
	35	Other people being aware of your private thoughts		♦										♦						
	36	Feeling others do not understand you or are unsympathetic		♦												♦				
	37	Feeling that people are unfriendly or dislike you													♦	(♦)				
	41	Feeling inferior to others													♦			♦		
	43	Feeling that you are watched or talked about by others		♦												♦				
	61	↔ Feeling uneasy when people are watching or talking about you									♦				♦	♦				
	68	Having ideas or beliefs that others do not share		♦							♦				♦					
	69	Feeling very self-conscious with others													(♦)					
	73	Feeling uncomfortable about eating or drinking in public		♦											♦					
	76	Others not giving you proper credit for achievements													♦	♦				
	83	Feeling that people will take advantage of you if you let them		♦											♦					
	88	Never feeling close to another person		♦																

SCL-90 items and dimensions			communities																	
			#1	#2	#3	#4	#5	#6	#7	#8	#9	#10	#11	#12	#13	#14	#15	#16	#17	#18
Acting-out hostility	11	Feeling easily annoyed or irritated		♦																
	24	Temper outbursts that you could not control				♦														
	63	Having urges to beat, injure or harm someone				♦														
	67	Having urges to break or smash things		♦		(♦)														
	74	Getting into frequent arguments				♦														
	81	Shouting or throwing things				♦														
Cognitive-performance deficits	9	Trouble remembering things					♦			♦										
	10	Worried about sloppiness or carelessness					(♦)													
	28	Feeling blocked in getting things done		♦																
	38	Having to do things very slowly to insure correctness		♦			♦													
	45	↔ Having to double-check what you do					♦			♦									♦	
	46	Difficulty making decisions					♦													
	55	Trouble concentrating		♦						(♦)										
	65	Having to repeat the same actions, such as touching, counting, washing				♦													(♦)	
	71	Feeling everything is an effort		♦																
Unscaled SCL-90 items	16	↔ Hearing voices that other people don't hear		♦		♦					♦									
	60	Overeating									♦									
	62	Having thoughts that are not your own		♦																
	77	Feeling alone even when you are with people		♦											♦					
	84	Having thoughts about sex that bother you a lot												♦			♦			
	85	The idea that you should be punished for your sins							♦					(♦)						
	87	The idea that something serious is wrong with your body																		♦
	89	Feelings of guilt												♦				♦		
	90	The idea that something is wrong with your mind									♦									

♦ Denotes symptoms belonging to a community (i.e., problem area)

(♦) Denotes 'stabilizing' symptoms within a community (i.e., *within* a problem area)

↔ Denotes 'communicating' symptoms between communities (i.e., *between* problem areas)

* Denotes symptoms that are not associated with any community (#5 Loss of sexual interest or pleasure - depression, and #78 Feeling so restless you couldn't sit still - anxiety)

Supplementary Methods. *Network Stability*

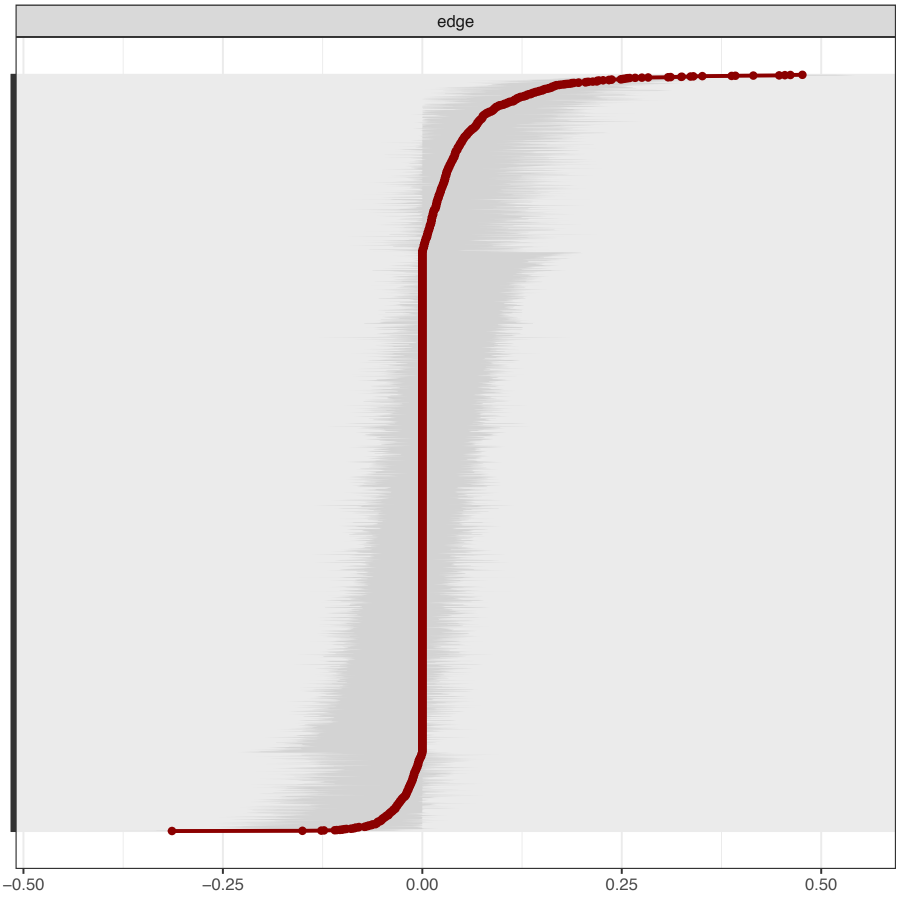

Figure S1. Bootstrapped edge weights. The red line depicts point estimates of the edge weights in the reported network, the grey bars represent 95% confidence intervals.

Bootstrapping results indicated good accuracy of the estimated network parameters, see Figure S1. In addition, we calculated the correlation stability coefficient (CS), which represents the maximum proportion of participants that can be dropped while maintaining results that correlate at least 0.7 with the results obtained on the complete sample. We specifically focused on the stability of the strength and number of connections a node has (i.e., degree centrality) as this is most relevant for our community detection analyses. With a CS of 0.52 the stability exceeded the proposed cut-off of 0.5, suggesting good stability.
